# Novel nanomicelle formulation to enhance bioavailability and stability of curcuminoids

**DOI:** 10.22038/ijbms.2019.32873.7852

**Published:** 2019-03

**Authors:** Mahdi Hatamipour, Amirhossein Sahebkar, Seyedeh Hoda Alavizadeh, Mahyar Dorri, Mahmoud Reza Jaafari

**Affiliations:** 1 Nanotechnology Research Center, Pharmaceutical Technology Institute, Mashhad University of Medical Sciences, Mashhad, Iran; 2 Department of Medicinal Chemistry, School of Pharmacy, Mashhad University of Medical Sciences, Mashhad, Iran; 3 Biotechnology Research Center, Pharmaceutical Technology Institute, Mashhad University of Medical Sciences, Mashhad, Iran; 4 Neurogenic Inflammation Research Center, Mashhad University of Medical Sciences, Mashhad, Iran; 5 Department of Pharmaceutical Nanotechnology, School of Pharmacy, Mashhad University of Medical Sciences, Mashhad, Iran

**Keywords:** Biological availability, Curcuminoid, Drug stability, Micelle, Pharmacokinetics

## Abstract

**Objective(s):**

Curcuminoids, comprising curcumin, demethoxycurcumin (DMC) and bisdemethoxycurcumin (BDMC), are bioactive phytochemicals with numerous pharmacological effects. Oral biological availability of curcuminoids is low due to the low aqueous solubility and rapid metabolism. This study aimed at fabricating a nanomicellar curcuminoid formula with enhanced pharmacokinetic properties.

**Materials and Methods:**

Curcuminoids nanomicelles were prepared and characterized regarding particle properties, stability, release profile and pharmacokinetic parameters.

**Results:**

Encapsulation efficiency of curcuminoids in nanomicelles were 100%. Particle size analysis demonstrated a mean size of around 10 nm that remained stable for 24 months. Dissolution test showed the complete dissolution of encapsulated curcuminoids from nanomicelles within 20 min while the free curcuminoids were poorly dissolved (approximately 7% after 60 min). The results of long-term (24 months) and accelerated (6 months) stability studies showed no changes in the size and content of nanomicelles. The release studies in simulated gastric fluid (SGF) and simulated intestinal fluid (SIF) showed no release of curcuminoids for at least 4 hours. *In vivo* study in BALB/c mice showed improved pharmacokinetic parameters including maximum plasma concentration (C_max_) and time to reach the maximum concentration (T_max_) with nanomicelles as compared to free curcuminoids and two other commercial products. T_max_ for all the three curcuminoid components was observed 30 min following oral administration. AUC of nanomicellar curcuminoids was 59.2 times more than free curcuminoids.

**Conclusion:**

These data indicated that nanomicelles could improve solubility, oral bioavailability and also the stability of curcuminoids. Thus, they merit further investigation for enhancing pharmacological effects of curcuminoids.

## Introduction

Curcuminoids are dietary polyphenols extracted from the dried rhizomes of *Curcuma longa* L. (turmeric), and comprise curcumin, demethoxycurcumin (DMC) and bisdemethoxycurcumin (BDMC). Curcuminoids are among the most widely studied natural products owing to their promising biological and pharmacological activities (1). Notably, curcumin is considered as a, generally recognized as safe (GRAS), compound by the United States Food and Drug Administration. Previous studies have revealed modulatory effects of curcuminoids on numerous biomolecules, some of which serving as key elements in the pathways regulating inflammation, oxidative stress, immune response and cellular proliferation and homeostasis (2, 3). In line with the molecular findings, *in vivo* and clinical studies have confirmed the antioxidant (4), anti-inflammatory (5, 6), anti-tumor (7, 8), analgesic (7), anti-arthritic (6), immunoregulatory (7), and lipid-modifying (6) activities of curcuminoids relevant to the treatment of human diseases. However, biological activities of curcuminoids in *in vivo* studies and clinical settings are hampered by the low oral biological availability of these phytochemicals (9). The low systemic biological availability following oral ingestion of curcuminoids is due to the low aqueous solubility, instability at physiological pH, as well as rapid metabolism and clearance (9). Thus, numerous attempts have been made to improve the pharmacokinetic characteristic of curcuminoids to enhance oral biological availability and consequently pharmacological effects (9, 10). 

In order to increase the solubility and improve stability and oral biological availability of poorly water-soluble compound, several strategies have been suggested. One is using nano-micelles that have emerged as efficient tools for the encapsulation of drugs with low aqueous solubility (11). The core–shell structure of micelles prevents the penetration and presence of water in its inner core. This key feature of micelles create a suitable environment for the encapsulated drug in comparison with the free drug (11). Some advantages presented by micelles as drug carriers, including easy development, affordable costs, facilitated transport of cargo across biological barriers, improved solubility in aqueous media including unstirred water layer of the intestine, controlled release profile, and protection against degradation (12). 

In this study, we report on the characterization and pharmacokinetic properties of a nanomicellar formulation of curcuminoids as compared to free curcuminoids and two commercial products. 

## Materials and Methods

***Materials***

Curcuminoids were purchased from Sami Lab Limited (Bengaluru, Karnataka, India) and contained 79.4% curcumin, 17.6% DMC and 3% BDMC. Content of total curcuminoids was 95.17%. Curcuminoids reference standards (curcumin, DMC and BDMC) were of USP reference standards and purchased from Rockville (MD, USA). HPLC grade tetrahydrofuran (THF) was purchased from Scharlau Co. (Barcelona, Spain). Citric acid was obtained from Merck Co (Merck Millipore, Massacheusetts, USA). Ultrapure water for chromatography was obtained using a Simplicity 185 Water Puriﬁcation system (Millipore, Bedford, MA, USA). Hard gelatin capsules (size 00) to fill the curcuminoids powder were obtained from the lab of School of Pharmacy, Mashhad University of Medical Sciences (Mashhad, Iran).

For comparison we used two commercial products of curcuminoids, one from Europe (CP1) and the other one from North America (CP2). These commercial products are frequently used as a curcuminoids supplement in Europe and North America. All other chemicals were of reagent grade and were used as received.

***Preparation of curcuminoid nanomicelles***

Curcuminoids nanomicelles were prepared using GRAS excipients (13-15).

***Particle size and morphology analysis***

The measurement of the particles size of nanomicelles was performed using dynamic light scattering (Zeta sizer, Malvern, UK). The light source was a diode pumped solid-state laser with a wavelength of 633 nm and a scattering angle of 90 ^o^. Size measurements were carried out in triplicate and the average of recordings was reported. The size of particles was also measured during long-term stability study and accelerated condition for 6 months. Samples were taken at 0, 3, 6, 9, 12, 15, 18, 21 and 24 months for long-term [25 ^°^C ± 2 ^°^C, 60% relative humidity ± 5%] and at 0, 1, 2, 4 and 6 months for accelerated condition [40 ^°^C ± 2 ^°^C, 75% relative humidity ± 5%] studies.

***Characterization of nanomicelles using transmission electron*** ***microscopy (*TEM*) by negative staining***

Diluted (1 to 100 in water) nanomicelles (20 µl) were deposited onto carbon coated copper grid. After 1 minute, the excess nanomicelles was removed by filter paper (Whatman®) and stained with 20 µl of 1 % sodium phosphotungstate pH 7.0 for 2 min. Then, the excess stain was removed by filter paper (Whatman®), the samples were viewed and photographed with a Phillips CM100 electron microscope (Philips/FEI Corporation, Eindhoven, Holland).

***CMC of the nanomicelles***

Critical micelle concentration (CMC) is known as the concentration of surfactants upon which the micelles are automatically formed. To determine the CMC of nanomicelles in distillated water, the acetone solution containing iodine (15 µl from 16.3 mg/ml) were added to 2 ml of various concentration of nanomicelles suspension (0.125-10 mg/ml) in distilled water. The absorption intensity was plotted against log (concentration). With a sharp increase in absorbance measurement using UV–VIS spectra (UV-160A Shimadzu) at λ_max_=369 nm, the CMC can be verified (16, 17). 

***Curcuminoids analysis by HPLC***

Determination of curcuminoids was carried out on a Shimadzu chromatographic system (Shimadzu, Kyoto, Japan) equipped with Solvent Delivery Unit LC-20A pump and UV-VIS detector SPD-20A according to the USP35. Curcuminoids reference standards (curcumin, demethoxycurcumin and bisdemethoxycurcumin) were of USP reference standards and purchased from Rockville (MD, USA). Samples were injected over a Rheodyne^®^ Model 3725i valve with a ﬁxed loop of 20 µl, and analyzed at 420 nm. Analyses of samples were performed on a reverse-phase C18 column (5 µm, 150 mm×4.6 mm, Symmetry, Waters, USA) at ambient temperature. The mobile phase consisted of HPLC-grade deionized water (containing 1 mg/ml citric acid) and tetrahydrofuran [65:35 (v/v)]. All analyses were carried out with a flow rate of 1.0 ml/min. 

**Figure 1:**
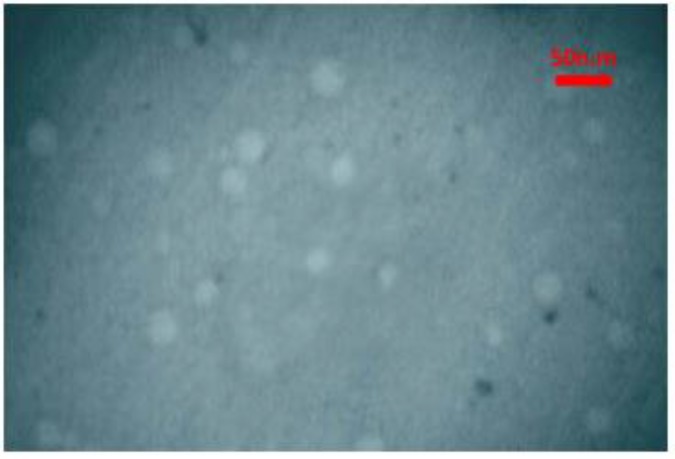
TEM image of nanomicelles. Diluted nanomicelles (20 µl) were deposited onto carbon coated copper grid. After 1 minute, the excess nanomicelles was removed by filter paper (Whatman®) and stained with 10 µl of 1 % sodium phosphotungstate pH 7.0 for 2 min. The samples were photographed with a Phillips CM100 electron microscope (Philips/FEI Corporation, Eindhoven, Holland)

**Figure 2:**
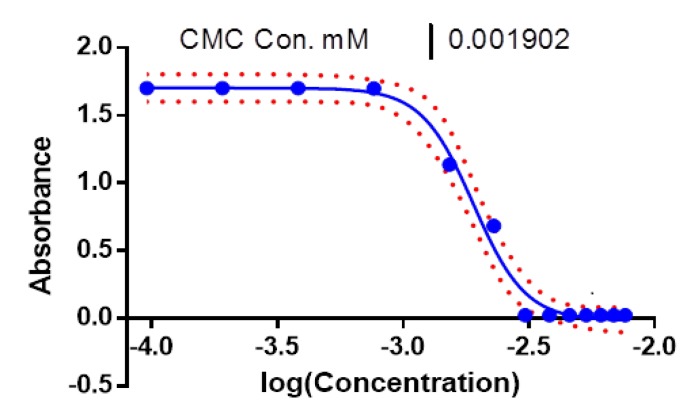
Critical micelle concentration (CMC) as a function of log concentration of nanomicelles. To determine the CMC of nanomicelles in distillated water, the acetone solution containing iodine (15 µl from 16.3 mg/ml) were added to 2 ml of various concentration of nanomicelles suspension (0.125-10 mg/ml) in distilled water. The absorption intensity was plotted against log (concentration). With a sharp increase in absorbance measurement using UV–vis spectra (UV-160A Shimadzu) at λ_max_=369 nm, the CMC can be verified

**Figure 3:**
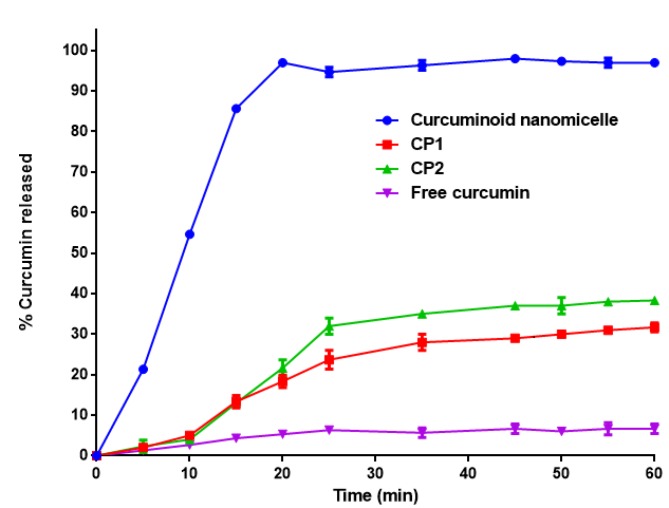
Release profile of curcuminoids according to dissolution test of USP35 (mean±SD, n=3). Dissolution of curcuminoids were determined in 900 ml water containing 1% sodium lauryl sulfate at 37.0 ± 0.5 °C at 100 rpm. Samples were obtained at 5, 10, 15, 20, 25, 30, 35, 40, 45, 50, 55 and 60 min and were passed through a 0.22 µm filter, diluted with mobile phase, and subjected to HPLC

CP1: commercial product 1; CP2: commercial product 2

**Figure 4:**
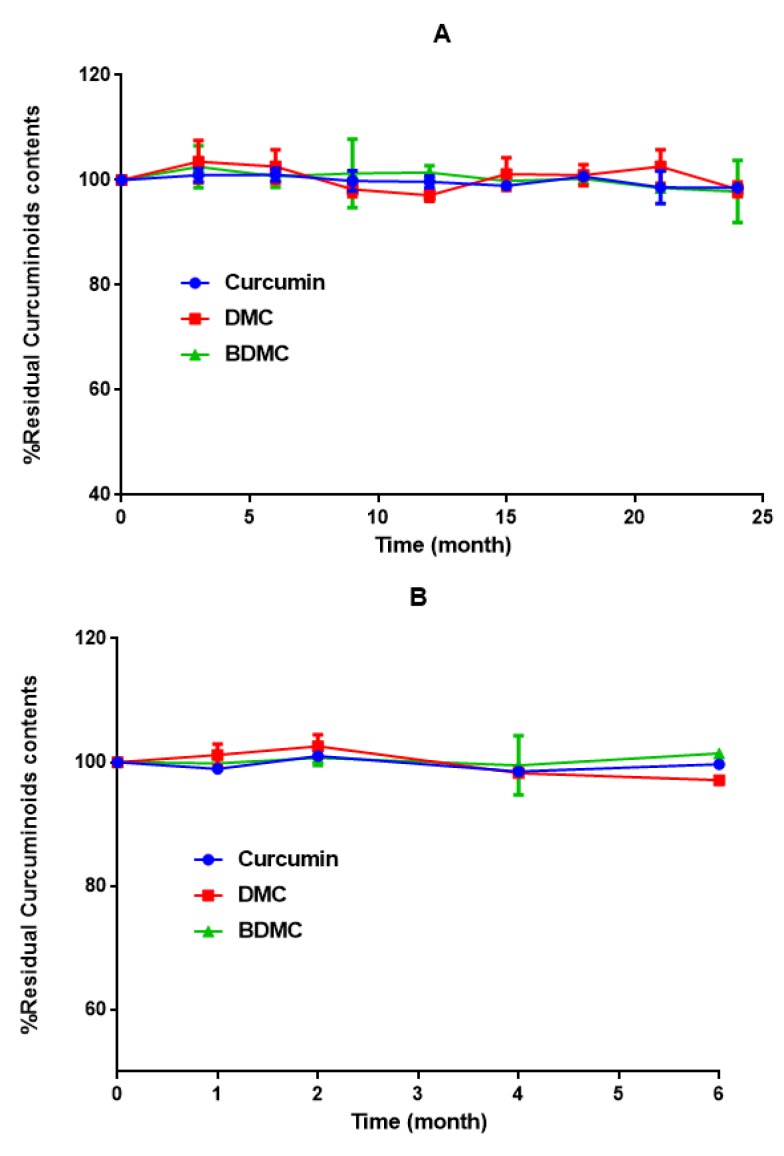
Long-term (A) and accelerated stability (B) studies of nanomicellar curcuminoid formula according to the ICH guidelines (2003) code Q1A(R2). Nanomicelles were stored in tube containers preserved from light. Samples were taken at 0, 3, 6, 12, 15, 18, 21 and 24 months for long-term [30 °C ± 2 °C, 65 % relative humidity ± 5%] and at 0, 1, 2, 4 and 6 months for accelerated condition [40 °C ± 2 °C, 75% relative humidity ± 5%] studies. Nanomicelles diluted with the HPLC mobile phase were then injected to HPLC column in triplicate

DMC: demethoxycurcumin; BDMC: bisdemethoxycurcumin

**Figure 5:**
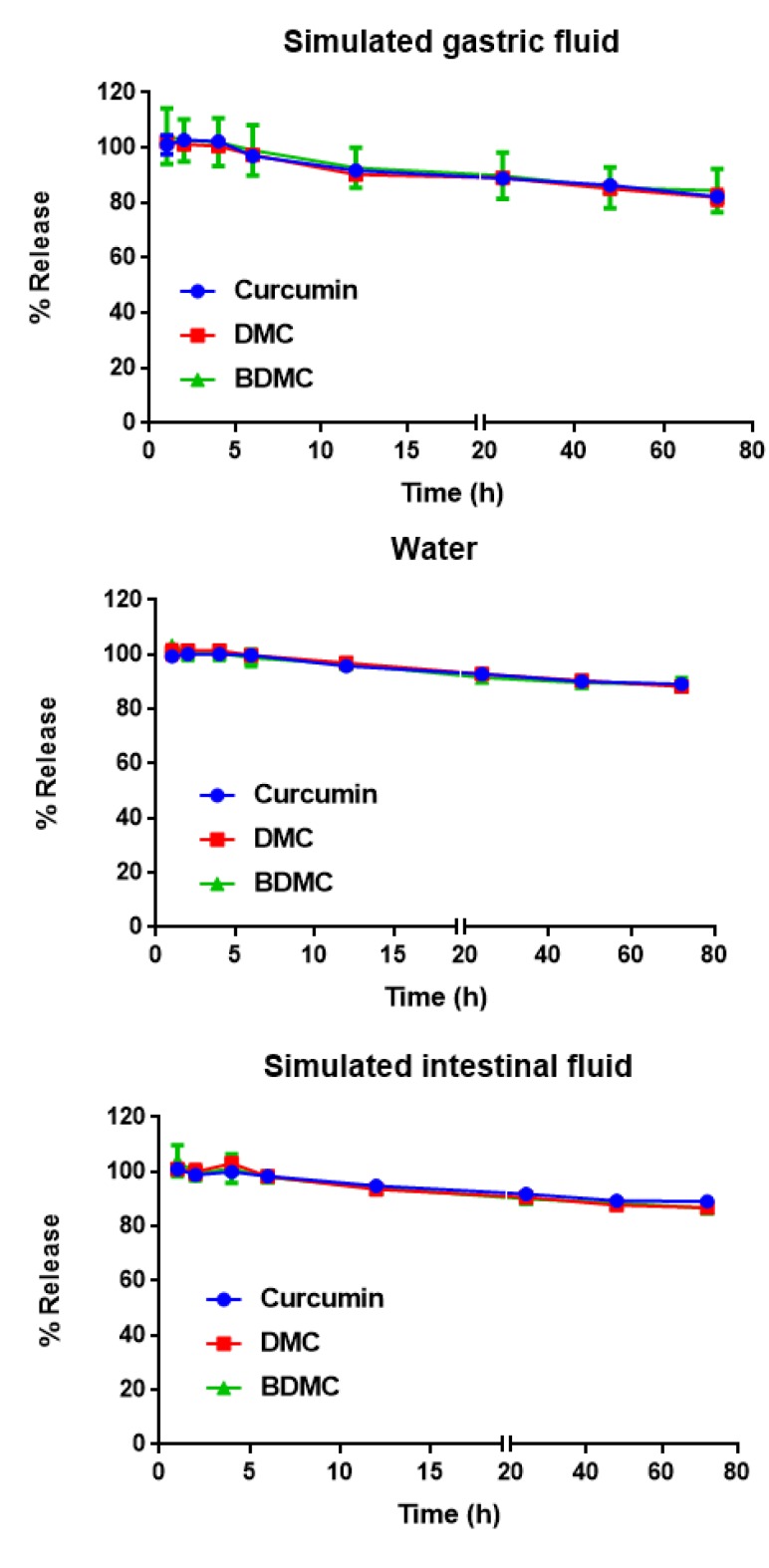
Release profiles of curcumin, demethoxycurcumin (DMC) and bisdemethoxycurcumin (BDMC) in simulated gastric fluid (SGF) (A) and simulated intestinal fluid (SIF) (B). Data represent the means ± SD (n=3). Nanomicelles were diluted in the above mentioned media (1:10), followed by sampling at 0, 1, 2, 4, 6, 12, 24, 48 and 72 hr time points and curcuminoids concentration were determined using HPLC

**Figure 6:**
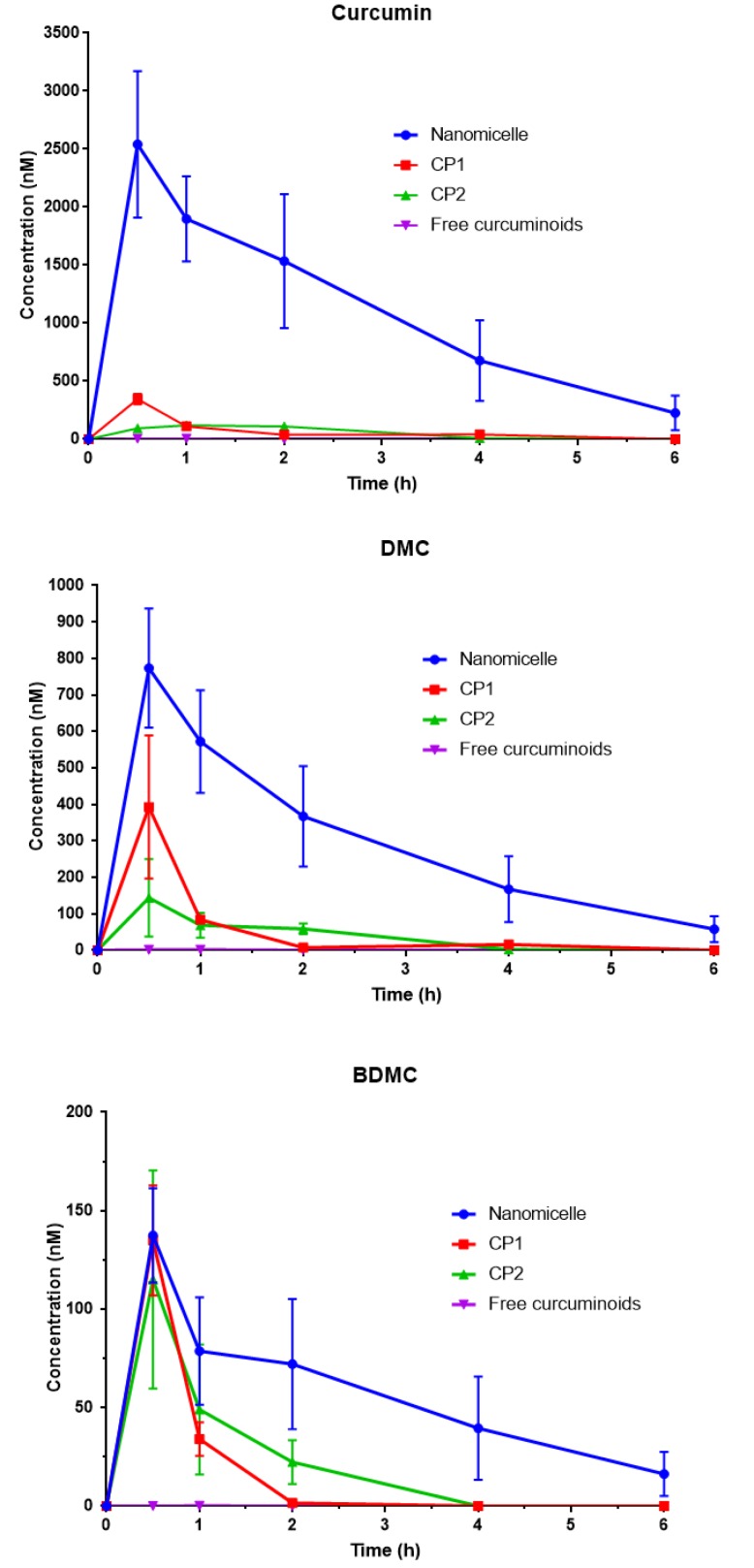
Concentration of curcumin, demethoxycurcumin (DMC) and bisdemethoxycurcumin (BDMC) in mouse plasma after a single oral administration of: nanomicellar, commercial product 1 (CP1), commercial product 2 (CP2) and free curcuminoids (35 mg/mouse). Data are expressed as mean±SD (n=3). Mice received a single dose (35 mg) of curcuminoids via oral gavage. Blood samples were obtained at 30 min, 1, 2, 4 and 6 h after dosing via heart puncture. Plasma was separated by centrifugations and deproteinated using methanol precipitation and was then injected to the HPLC system

**Figure 7:**
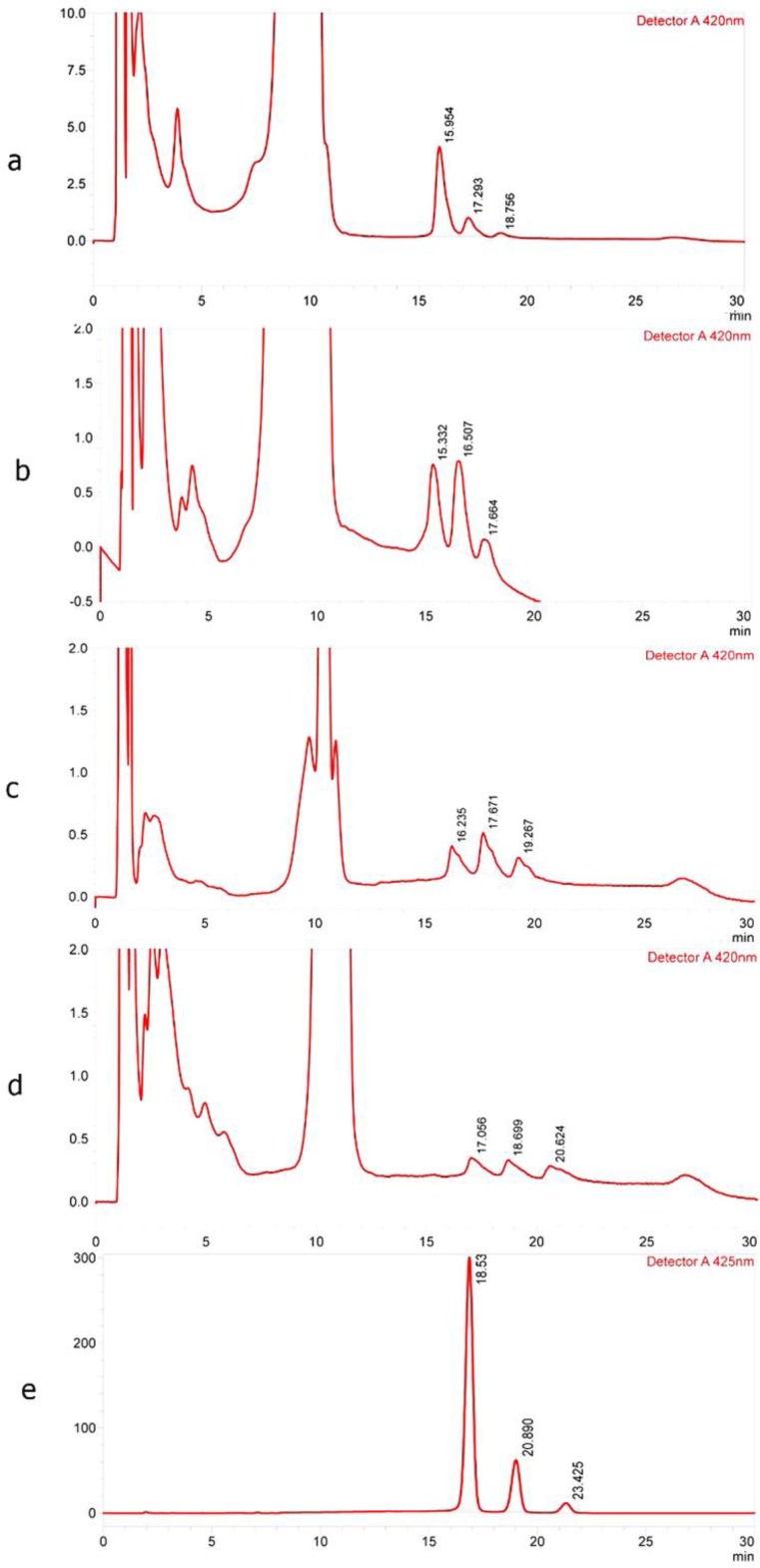
Typical chromatogram of curcuminoids in plasma after dosing (a) Sinacurcumin, (b) CP1, (c) CP2, (d) pure curcuminoids and (e) chromatogram corresponding to standard

**Table 1:** The size of nanomicelles during the long-term stability study for 24 months and accelerated condition for 6 months

Time (month)	z-average (nm)
0	9.9±0.1
3	9.2±0.1
6	9.6±0.1
9	9.7±0.1
12	9.2±0.1
15	9.4±0.2
18	9.2±0.1
21	9.4±0.2
24	10.2±0.1
Time (month)	z-average (nm)
0	9.1±0.1
1	8.8±0.1
2	9.7±0.1
4	10.8±0.1
6	9.0±0.1

**Table 2:** Pharmacokinetic parameters of curcumin, DMC and BDMC following oral administration of nanomicelles, commercial products 1 (CP1) and 2 (CP2) and also their free form in mice (n= 3)

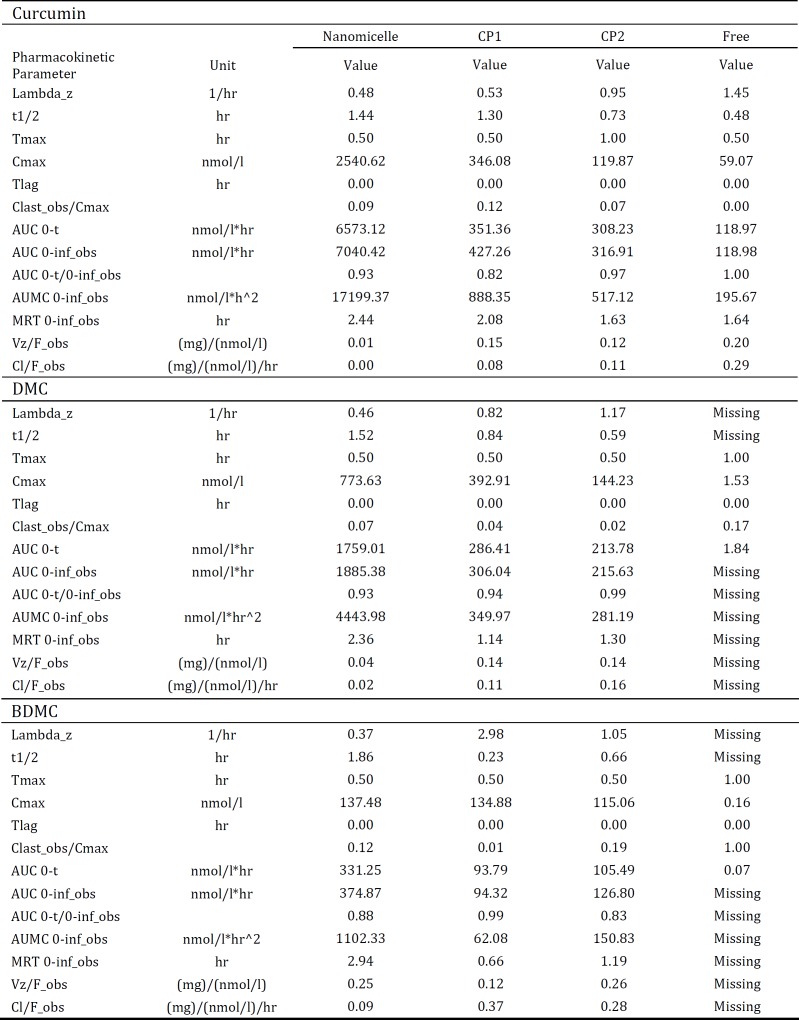

***Drug loading and encapsulation efficiency***

Determination of the amount of drug encapsulated in the nanomicelles was performed using ultrafiltration method on centrifugal filter units with a molecular weight cut-off of 12 kDa (Merck Millipore; Massachusetts, USA). Briefly, nanomicelles were diluted with dextrose 5% in a 1:9 ratios, and added into a centrifugal filter unit which was then centrifuged at 4000 g for 30 min. After centrifugation, the amount of free curcuminoids in the flow-through was assayed using HPLC. Unfiltered nanomicelles were heated, dissolved in DMSO and sonicated to extract the drug. The resulting solution was then diluted with methanol and drug concentration was measured using HPLC. Encapsulation efficiency was calculated as follows: 

***Dissolution test***

The dissolution properties of nanomicellar curcuminoids, free curcuminoids, CP1 and CP2 were investigated according to USP35 using apparatus 2 of the USP (paddle apparatus, PTWS3F, PharmaTest, Hainburg, Germany). Dissolution of curcuminoids were determined in 900 ml water containing 1% sodium lauryl sulfate (SLS) at 37.0 ± 0.5 ^°^C and the release was investigated at 100 rpm. Samples (5 ml, equivalent to 1 g) were obtained at 5, 10, 15, 20, 25, 30, 35, 40, 45, 50, 55 and 60 min time points for analysis, and medium solution was immediately replaced after each sampling. Samples were passed through a 0.22 µm filter (Sartorius AG, Goettingen, Germany), diluted with mobile phase, and subjected to HPLC (Shimadzu, Japan).

***Stability studies of curcuminoids according to ICH***

Stability of nanomicelles was assessed according to the ICH (The International Council for Harmonisation of Technical Requirements for Pharmaceuticals for Human Use) guidelines (2003) code Q1A(R2) (stability testing of new drug substances and products). Nanomicelles were stored in impenetrable tube containers and preserved from light. Samples were taken at 0, 3, 6, 12, 15, 18, 21 and 24 months for long-term [30 ^°^C ± 2 ^°^C, 65 % relative humidity ± 5%] and at 0, 1, 2, 4 and 6 months for accelerated condition [40 ^°^C ± 2 ^°^C, 75% relative humidity ± 5%] studies. Twenty µl of nanomicelles (diluted with the HPLC mobile phase to a final concentration of 30 to 50 µg/ml) was then injected to HPLC column in triplicate, to determine the content of curcuminoids in each sample.

***Release studies in*** ***simulated gastric fluid and simulated intestinal fluid***

The release profile of curcuminoids nanomicelles was investigated in simulated gastric fluid (SGF) and simulated intestinal fluid (SIF) in order to determine the stability of nanomicelles in SGF and SIF. To prepare the SGF, hydrochloric acid solution (0.2 N, 39 ml) was added to sodium chloride solution (0.2 N, 250 ml), 600 ml deionized water (DW) was added and the pH was adjusted at 2.2, then the final volume was adjusted to 1000 ml with DW. SIF was prepared by dissolving KH2PO4 (6.8 g) in 250 ml of DW. This solution was mixed with NaOH solution (0.2 N, 77 ml), 600 ml deionized water (DW) was added and the pH was adjusted at 6.8, then the final volume was adjusted to 1000 ml with DW.

Nanomicelles were diluted in the SGF and SIF in a ratio of 1:10 and incubated at 37±1.0 ^°^C, followed by sampling at 0, 1, 2, 4, 6, 12, 24, 48 and 72 hr time points. To separate the released curcuminoids, the samples filtered through 0.22 µm microbial filter to separate the precipitated curcuminoids. Since the solubility of curcuminoids in water is too low, they precipitate right after the disruption and release from nanomicelles. Then the determination of curcuminoids in purified nanomicelles was accomplished by HPLC. For HPLC determinations, 20 µl of nanomicelles (diluted with the HPLC mobile phase to a final concentration of 30 to 50 µg/ml) was injected to HPLC column in triplicate. 

***Pharmacokinetic*** ***study***

Female BALB/c mice (weight: 20±2 g) were obtained from the Pasteur Institute of Tehran, Iran. All animal studies were done in compliance with the Institutional Ethical Committee and Research Advisory Committee of Mashhad University of Medical Sciences (dated May 2, 2012; proposal code 910042). Animals were acclimatized to the laboratory conditions (temperature of 25 ± 2 °C and natural light/dark cycles) for at least 24 hr before oral administration. Mice were starved for 12 hr prior to the experiment. Mice were randomly assigned to either of the following groups: the nanomicellar curcuminoids, commercial product 1 (CP1), commercial product 2 (CP2) or free curcuminoids groups (n=3 in each group). Each mouse received a single dose (35 mg) of curcuminoids via oral gavage. Blood samples (0.5 ml) were taken at 30 min, 1, 2, 4 and 6 hr after dosing via heart puncture and were transferred into heparinized tubes. Plasma was separated by centrifugations at 10000 *g* for 10 min and stored at -20 ^°^C prior to analysis. For analysis, plasma samples were first deproteinated using methanol precipitation method. The clear methanolic layer was separated by centrifugation at 12000 g for 10 min and methanol was then evaporated under a gentle stream of nitrogen. The residue was dissolved in the mobile phase and 20 μl aliquots were injected to the HPLC system. Concentration of each curcuminoids versus time and the pharmacokinetic parameters analysis were performed with PKsolver (An add-in program for pharmacokinetic data) (18).

***Statistical analysis***

All statistical analyses were performed using Graph Pad Prism 6 Software. One-way ANOVA and two-way ANOVA followed by Tukey’s post-test were done to assess the significance among various groups. Results with *P*<0.05 were considered significant.

## Results

***Nanomicelles characterization***

The encapsulation efficiency of curcuminoids in nanomicelles was calculated as 100%. The mean diameter of nanomicelles was 9.5±0.1 nm, according to dynamic light scattering (Table 1). The size of nanomicelles remained constant during long-term stability study for 24 months and 6 months accelerated condition (Table 1). The morphology of nanomicelles using TEM, revealed that nanovesicles have spherical-like shapes, as expected for micelles (Figure 1). 

CMC of nanomicelles was calculated to be 0.0019 mM (Figure 2).

***In vitro dissolution study***

Dissolution study was carried out according to USP35. As shown in Figure 3, 100% of the encapsulated curcuminoids in nanomicelles were dissolved in water containing 1% SLS after 20 min while free curcuminoids were sparingly soluble (approximately 7% release after 60 min). Only 22 and 25% of CP1 and CP2 were dissolved after 60 minutes, respectively.

***Stability test according to ICH***

No changes were observed in the size (Table 1) and content of curcuminoids in nonomicelle formulation in either long-term or accelerated stability studies (Figure 4). 

***Release studies in simulated gastric fluid and simulated intestinal fluid***

The level of curcumin, DMC and BDMC did not change significantly in the first 4 hr (~100%) in SGF and SIF and reached to 98% of original concentration at 0 time (Figure 5). However, curcumin, DMC and BDMC concentrations were reduced to 82, 81 and 84%, respectively, following 72 hr incubation in SGF. The stability of nanomicelles were higher in SIF as compared to SGF. In SIF, the concentrations of curcumin, DMC and BDMC decreased from 98% at 6 hr, to 89, 87 and 87%, after 72 hr, respectively (Figure 5).

***Pharmacokinetic study***

Total curcuminoids levels in mice plasma were calculated following oral gavage using HPLC. The concentration-time profiles after oral gavage of curcuminoids-loaded nanomicelles, CP1 and CP2, as well as free curcuminoids at an equal dose of 35 mg/mouse were compared (Figure 6 and 7). There was a clear decrease in the level of curcuminoids over time in both nanomicelles and CPs. Nevertheless, the rate of reductions in curcuminoids levels in nanomicelles treated mice were much lower. As illustrated in Figure 6, at 0.5 hr post-treatment, the plasma concentration of curcumin was significantly higher in mice received nanomicelles than CP1 (*P*<0.05) and CP2, and free curcumin (*P*<0.01), respectively. At 1 hr, the difference between nanomicelles and CPs was much more dramatic (*P*<0.001). The level of plasma curcumin 2 hr post–treatment was still significant for nanomicellar formulation (*P*<0.05) compared to other ones, however, to a much lower extent. For the DMC, the same trend was kept. At 1 hr post-treatment, the DMC plasma level were reduced dramatically for CP1, CP2 and free curcuminoids, which was significantly lower than that of nanomicelles (*P*<0.01). With nanomicelles, the plasma level of BDMC, significantly increased as compared to free curcuminoids (*P*<0.05) (Figure 6). 

Pharmacokinetic parameters such as maximum plasma concentration (Cmax) and time to reach the maximum concentration (T_max_) were significantly improved in the nanomicellar versus free curcuminoids (Table 2). Following oral administration of nanomicelles, the curcumin C_max_ of approximately 2540.62 nmol/l was reached after 30 min, while the values were 346.08 and 59.07 for CP1 and free curcumin, respectively. Free curcumin was rapidly metabolized, resulted in a short t ½ of 0.48, as compared to nanomicelle (t ½: 1.44 hr). T_max_ for all three curcuminoids components, i.e. curcumin, DMC and BDMC achieved at 30 min following administration of nanomicelles. Nanomicelle formulation significantly increased the AUC 0-t and AUC 0-inf of curcumin as compared to CP1, CP2 free curcumin. The AUC 0-inf of curcumin for nanomicelles was 16.5, 22.3 and 59.2 times more than CP1, CP2 and free curcuminoids. The biological availability of other ingredients including DMC and BDMC was also superior following administration of nanomicelles as compared to their free form or CPs (Table 2). 

## Discussion

The present study described physicochemical characteristics and pharmacokinetic profile of a nanomicellar carrier system of curcuminoids. Curcuminoid nanomicelles were found to have a considerably higher aqueous solubility and systemic biological availability compared to the free curcuminoids and commercial products. The nanomicelles also showed a constant dissolution profile and favorable long-term stability. These improvements could be attributed to the unique core-shell structure of micelles accommodating hydrophobic cargo (curcuminoids) in their core whilst the hydrophilic shell contributes in solubilizing particles in the aqueous media. Further, micellar systems could physically prevent degradation and inactivation of their cargo in biological environments such as SFG and SIF. This was indeed confirmed by the present findings on the stability of curcuminoids in different media, as well as the long-term and accelerated stability tests. Nanomicelles could also pass – and transport their cargo – through enterocytes via direct endocytosis due to their small size (19).

Other studies have reported on an improved solubility and pharmacokinetic characteristic of curcumin nanomicellar system employing methoxy poly (ethylene glycol)-block polycaprolactone diblock copolymers (20), Pluronics P121 and F68 (21), methoxy poly(ethylene oxide)-b-poly(ε- caprolactone) (22) and Pluronic F127 (23).

The formulation of nanomicelles is somehow that the developed nanomicelles are stable in SGF and SIF for at least 4 hours, as shown in Figure 5. This time (4 hours) is well enough for nanomicelles to pass the stomach and reach to the intestine, where they can absorb with different mechanisms. Our data on pharmacokinetic parameters and plasma concentration of curcumin is also a good proof to the stability of nanomicelles in SGF and SIF. Plasma concentration of curcumin was significantly higher in nanomicelles compared to the free curcumin, confirming the high absorption of nanovesicles form the gastrointestinal tract. 

*In vivo* pharmacokinetic study showed a rapid elevation in the plasma concentration of curcumin, DMC and BDMC in the first 30 min (T_max_) after dosing, reaching maximum concentrations of 2540, 773 and 137 nM, respectively. These data suggest that oral absorption of nanomicellar curcuminoids were significantly increased compared to free curcuminoids, and CP1 and CP2.

Several mechanisms are involved in the enhanced absorption of nanomicellar curcuminoids. First, direct uptake of nanoparticles from the GI tract improves bioavailability of encapsulated curcuminoids; as absorption rate in the GI tract highly depends on the particle diameter (24). Secondly, surfactants incorporated in the structure of nanocarriers increase the permeability of intestinal brush border and plasma membranes to drug molecules. Finally, increased stability and prolonged circulation half-life following encapsulation could further enhance plasma levels of curcuminoids. In this context, degradation of curcuminoids by bacteria and enzymes in the GI tract has been demonstrated, while nanomicellar encapsulation can decrease these metabolic breakdowns (25). 

An important feature of the formula presented in this study is the nanoencapsulation of triple curcuminoids complex. Previous studies have mainly dealt with the improvement of pharmacokinetic properties of curcumin. Though the pharmacological benefits of curcumin are well-documented, it is important to note that curcumin is not the only bioactive curcuminoid. Recent studies have revealed that other curcuminoid structures, mainly DMC and BDMC, possess promising pharmacological effects that in some instances are even greater than curcumin (26). Therefore, obtaining a single formulation incorporating and improving the pharmacokinetic profile of all the three major curcuminoids could result in achieving a greater therapeutic response.

## Conclusion

In summary, this study presented data on the successful fabrication of a nanomicellar formula with enhanced solubility, oral bioavailability and stability of all the three major curcuminoids i.e. curcumin, DMC and BDMC. Future studies are warranted to test if the enhanced bioavailability of nanomicellar curcuminoids could provide superior pharmacological effects over free curcuminoids.

This formulation has been commercialized with the trade name of SinaCurcumin by Exir Nano Sina Company (Tehran, Iran), and is utilized as curcuminoids supplement.

## Conflicts of Interest

The authors report no declarations of interest.
